# Validity and reliability of the Chinese version of human–robot interaction self-efficacy scale in Chinese adults

**DOI:** 10.1186/s41155-024-00324-z

**Published:** 2024-09-26

**Authors:** Huixin Gao, Wei Wang, Chengli Huang, Xinru Xie, Duming Wang, Wei Gao, Jie Cai

**Affiliations:** 1 Department of Psychology, Zhejiang Sci-Tech University, 928 2nd Street, Xiasha Higher Education Park, Hangzhou, Zhejiang Province China; 2 Naval Medical Center of PLA, Naval Medical University, Shanghai, China; 3 School of Psychology, University of Southampton, Southampton, UK

**Keywords:** Self-efficacy, Human–robot interaction, Reliability, Validity

## Abstract

**Background:**

With the fast-paced advancements of robot technology, human–robot interaction (HRI) has become increasingly popular and complex, and self-efficacy in HRI has received extensive attention. Despite its popularity, this topic remains understudied in China.

**Objective:**

In order to provide a psychometrically sound instrument in China, this study aimed to translate and validate the Self-Efficacy in Human–Robot Interaction Scale (SE-HRI) in two Chinese adult samples (N1 = 300, N2 = 500).

**Methods:**

The data was analyzed by SPSS 26.0 and Amos 24.0. Item analysis and exploratory factor analysis were conducted using Sample 1 data. Confirmatory factor analysis, criterion-related validity analysis, and reliability analysis were then performed using Sample 2 data.

**Results:**

The results revealed that the Chinese SE-HRI scale consisted of 13 items in a two-factor model, suggesting a good model fit. Moreover, general self-efficacy and willingness to accept the use of artificial intelligence (AI) were both positively correlated with self-efficacy in HRI, while negative attitudes toward robots showed an inverse correlation, proving the Chinese SE-HRI scale exhibited excellent criterion-related validity.

**Conclusion:**

The Chinese SE-HRI scale is a reliable assessment tool for evaluating self-efficacy in HRI in China. The study discussed implications and limitations, and suggested future directions.

## Introduction

With the advancement of artificial intelligence (AI), AI-driven robotic applications are making significant strides and gradually undertaking roles traditionally performed by humans, particularly in some challenging tasks. For example, deep-sea marine robots play a crucial role to assist humans in scientific research, resource exploration, rescue missions, and environmental monitoring in the deep sea (Chen et al., 2023). Gynecological surgical robots can be precisely controlled by robotic arms to perform highly precise manipulations and reduced vision of localized tissues, enabling surgeons to perform operations with greater precision and less trauma to patients (Suzuki et al., 2023). And industrial robots are employed on automated production lines to replace or assist manual labor in heavy, hazardous, or repetitive tasks (Duan et al., 2023). Moreover, robots are entering public and private spaces in some cities, engaging with humans directly. For instance, social robots offer companionship and interactive experiences for children (Van Straten et al., 2023), service robots provide customers with a variety of services and information to enhance their experience in hotels (Xu et al., 2023), and care robots deliver nursing assistance to the elderly (AboJabel & Ayalon, 2023). With the widespread application of robots, the interaction between humans and robots has grown increasingly pivotal and frequent. Consequently, comprehending human–robot interaction has become an imperative and pervasive issue for researchers.

Extensive research has demonstrated that self-efficacy become an indispensable element in the human–robot interaction (Hsu & Chiu, 2004; Latikka et al., 2019; Rahman et al., 2016). Self-efficacy refers to individuals’ evaluations of their capacity to efficiently plan and execute requisite actions for specific tasks or performances (Bandura, 1986), which has garnered considerable scholarly attention (Brunes et al., 2021; Cayır & Ulupınar, 2021; Emirza et al., 2021; FitzGerald et al., 2022; Li et al., 2023; Morales-Rodríguez & Pérez-Mármol, 2019; Nygaard et al., 2016; Van Zyl et al., 2022). With the progression of robotics, academic interest in self-efficacy within human–robot interaction has steadily risen (Adami et al., 2023; Huang et al., 2014; Liao et al., 2023; Mallik et al., 2023; Savela et al., 2022; Turja et al., 2019). Specifically, self-efficacy in human–robot interaction pertains to individuals' perceptions of their ability to use robots (Pütten & Bock, 2018). Previous researches has shown that self-efficacy in human–robot interaction predicts higher trust in robots (Oksanen et al., 2020), and increasing self-efficacy can promote operators' willingness to use robots (Hampel et al., 2023). Turja et al. (2019) demonstrated that self-efficacy in human–robot interaction serves as a constructive foundation for new technologies acquisition in healthcare work. Specifically, the higher the level of self-efficacy in human–robot interaction, the more confident caregivers were in acquiring proficiency in robot usage. Likewise, Adami et al. (2023) discovered that fostering trust in the robot and its capabilities (e.g., self-efficacy) enabled workers to safely and effectively teleoperate robots on construction sites. Therefore, assessing self-efficacy in human–robot interaction has become a focal point in contemporary research.

Using broad scales to gauge self-efficacy in HRI may result in inaccurate assessments of individuals' capabilities to interact with robots (Pütten & Bock, 2018). Therefore, to mitigate potential misestimations of individuals' proficiency in interacting with robots, Pütten & Bock (2018) developed and validated the Self-Efficacy in Human–Robot Interaction Scale (SE-HRI). This scale is the first scale specifically designed to assess self-efficacy during human–robot interaction, identifying various aspects determining the quality of functioning in the context of human–robot interaction. For example, it focused participants' beliefs about how easily they could learn to use or control a robot (example items: “Robots are easy to control.”, “I could easily learn how to use a robot.”). Also, it addressed the customization of robots to suit individual needs (example items: “I could teach a robot to complete easy tasks.”, “I could do easy adjustments on a robot by myself.”). Moreover, it evaluated the perceived ability to comprehend the cause-and-effect relationship in a robot's behavior (example items: “If I would use a robot, I would always know how and why it behaves like it does.”). In sum, it has demonstrated robust psychometric properties (Pütten & Bock, 2018).

Amidst China's economic and technological development, the robot market is flourishing and booming, offering convenience, efficiency, and security to Chinese society. Chinese roboticists have made notablestrides in developing advanced robotic systems (Ding et al., 2018). For example, Qihoo 360 has developed Children Robots that can photograph, sing, and provide educational content based on big data and interactive voice feedback (Wang et al., 2019). Another noteworthy creation, Ubtech’s Alphal robot, showcased a dance performance at the Chinese New Year Gala (Wang et al., 2018). Additionally, Beijing Kangli Youlan’s commercial robot, named “Yoyo”, has made remarkable progress in various aspects. With its advanced capabilities in deep voice interaction, emotion recognition, movement control, and automatic obstacle avoidance, Yoyo is capable of providing shopping advice and supervising learning activities (Wang et al., 2018). Furthermore, Baidu launched the “Apollo Program” aimed at developing a car driver platform, addressing the challenges faced by traditional car manufacturers (Feng & Zhang, 2022). Consequently, an escalating number of companies and research groups engage in the competition to advance unmanned driving technology. Given these developments, it is necessary to explore self-efficacy in HRI within the Chinese context. However, to the best of our knowledge, this area field remains in a blank state in China, likely due to the absence of an effective assessment tool. Thus, revising the Chinese version of the SE-HRI scale has become an urgent priority. The SE-HRI scale distinguishes artificial intelligence robots from traditional computers, ensuring the specificity in self-efficacy assessment. Having been tested for reliability and validity in both English and German versions (Pütten & Bock, 2018), it holds the potential to become an important research tool in China.

In conclusion, this research assesses the reliability and validity of the SE-HRI scale within the context of Chinese culture. It will contribute to a deeper comprehension of individuals’ self-efficacy with technology products and provide a theoretical foundation for improving human experiences in technology applications. Furthermore, it has the potential to advance both research and practices in the field of human–robot interaction.

## Material and method

### Data source and participants

Data was collected via a Chinese questionnaire website (https://www.credamo.com/) in August 2023. Two sample groups were selected randomly for this study (*N1* = 300, *N2* = 500). Each participant was briefed on the objectives and procedures of this study before completing the survey. After completing the questionnaire, participants received a bonus of 2 CNY. The study was approved by the Institutional Review Board of the Institute of the Psychology.

Demographic features for Sample 1 and Sample 2 are presented in Table 1.

**Table 1:** Demographic characteristic of Sample 1 and Sample 2

		Sample 1 (*N* = 300)		Sample 2 (*N* = 500)	
Variables		N	Percent (%)	N	Percent (%)
Gender	Male	121	40.30	233	46.60
	Female	179	59.70	267	53.40
Age	Below 18	10	3.30	10	2.00
	19–25	242	80.70	251	50.20
	25–34	40	13.30	171	34.20
	35–54	8	2.70	54	10.80
	55–64	0	0.00	10	2.00
	Above 65	0	0.00	4	0.80
Education	High school and below	13	4.30	21	4.20
	Vocational high school	28	9.30	38	7.60
	Bachelors	186	62.00	324	64.80
	Masters	73	24.30	117	23.40

### Measures

The original version of the Human–Robot-Interaction Scale Self-efficacy (SE-HRI) comprises eighteen items. Participants were asked to rate these items on a six-point Likert scale (1 = *Strongly Disagree* to 6 = *Strongly Agree*). Higher scale scores indicate greater HRI self-efficacy. Following the recommendation of Regmi on translation/back-translation (Regmi et al., 2010), firstly, two postgraduates from the psychology department at Zhejiang Sci-Tech University translated SE-HRI individually. Afterward, researchers discussed the differences between the two versions, made corrections, and formed an initial Chinese version of SE-HRI. Then, three psychologists who are proficient in both Chinese and English re-translated it into English. Next, two researchers deliberated on distinctions between the original version of SE-HRI and the re-translated English version. Subsequently, we modified the language and wording of the initial Chinese version based on the differences in two different English versions. Finally, 10 postgraduates assessed the initial Chinese version for any ambiguous expressions. Following these steps, the Chinese version of SE-HRI was finalized.

### Criterion-related validity

To further substantiate the validity of the Chinese SE-HRI, General Self-Efficacy Scale (GSES), Willingness to Accept the Use of AI Devices Scale, and Negative Attitudes toward Robots Scale (NARS) were adopted to assess the criterion-related validity.

#### General Self-Efficacy Scale (GSES)

General self-efficacy refers to an individual's perception or belief in their ability to engage in adaptive behaviors when confronted with challenges (Schwarzer et al., 1997). The conceptualization of general self-efficacy is congruent with that of HRI self-efficacy (Latikka et al., 2019). The General Self-Efficacy Scale is designed to evaluate individuals' confidence in handling diverse tasks and situations (Luszczynska et al., 2005), whereas the HRI Self-Efficacy Scale addresses confidence in interacting with robots. Despite differing contexts, their fundamental concepts are analogous, suggesting that the general self-efficacy can serve as a pertinent criterion-related variable to validate SE-HRI. In the present study, the General Self-Efficacy Scale (GSES) is utilized, which includes 10 items. For example, “I can always manage to solve difficult problems if I try hard enough.” Participants were asked to rate the items on a 4-point Likert scale (1 = *Strongly Disagree* to 4 = *Strongly Agree*). The Cronbach’s α coefficient was 0.900.

#### Willingness to Accept the Use of AI Devices Scale

Willingness to Accept the Use of AI Devices refers to a customer's willingness for future service encounters with AI devices (Gursoy et al., 2019). This willingness can partially reflect a user's self-efficacy in HRI tasks. When users express their willingness to accept and use AI devices, it implies that their belief in successfully completing tasks through device interaction. In this study, we adopted the three items from the Willingness to Accept the Use of AI Devices Scale. Participants were asked to rate these items on a 5-point Likert scale (1 = *Strongly Disagree* to 5 = *Strongly Agree*). The Cronbach’s α coefficient was 0.629.

#### Negative Attitudes toward Robots Scale (NARS)

Negative Attitudes toward Robots encompass the negative attitudes and emotions that individuals hold toward robots (Nomura, Suzuki, et al., 2006). Originating from dissatisfaction or distrust in the robot's functionality, appearance, behavior, or interaction, these negative attitudes may lead to individuals' resistance to robot interaction, thus impacting cooperation with the robot, willingness to use it, as well as the sense of self-efficacy. In the realm of human–robot interaction, users' self-efficacy is closely related to their attitudes and emotions toward the robot. For instance, negative attitudes can diminish self-efficacy in interacting with the robots (Nomura, Kanda, et al., 2006). Therefore, utilizing the Robot Negative Attitude Scale as a validity measure contributes to a more comprehensive understanding of self-efficacy characteristics in human–robot interaction. In this study, we adopted Negative Attitudes toward Robots Scale (NARS) (Nomura, Suzuki, et al., 2006), which consists of 13 items. Participants were asked to rate these items on a 5-point Likert scale (1 = *Strongly Disagree* to 5 = *Strongly Agree*). The Cronbach’s α coefficient was 0.892.

### Data analysis

**Fig. 1:**
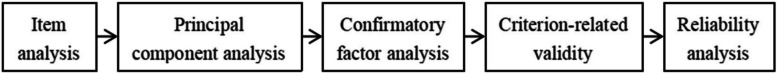
The process of data analysis

The data were analyzed using SPSS 26.0 and Amos 24.0. Item analysis and principal component analysis were conducted on Sample 1 data. Confirmatory factor analysis, criterion-related validity analysis, and reliability analysis were then performed on Sample 2 data. The whole data analysis process is shown in Fig. 1.

## Results

### Item analysis

First, the discriminability index method was used to examine each item difference between high (27%) and low (27%) groups based on the total score (see Table 2). The results showed that all the items exhibited significant differences between the high and low groups (*t* = 7.913 ~ 14.805, *p*s < 0.001), indicating high discriminability for all items (Costello & Osborne, 2005). Subsequently, the item-total correlation method was adopted to investigate the correlation between each item and the total score. Items with a correlation coefficient below 0.4 were considered for exclusion (Sheng et al., 2003), and the results revealed that the correlation coefficients for all 18 items and the total score ranged from 0.514 to 0.727, *p*s < 0.001, suggesting that no items required removal.

**Table 2:** Item description statistics and item analysis results (*N* = 300)

	Mean (*M*)	Standard Deviation (*SD*)	CR(*t*)	Item-total correlation(*r*)
1. I could set up a robot according to my wishes and my environment.	4.02	1.026	12.471*	.675**
2. I could get a robot to perform a specific task.	4.315	0.995	11.735*	.661**
3. I am familiar with technology; therefore, I think I could use a robot.	3.91	0.901	14.805*	.701**
4. I think I could adjust a robot the way that it could help me in my daily life.	4.56	0.9905	10.56*	.619**
5. It is easy to use a robot.	3.79	1.034	11.022*	.619**
6. If I should solve a problem with the assistance of a robot, I could do that.	4.305	1.014	7.913*	.514**
7. To achieve a specific goal with the assistance of a robot will not be a problem for me.	4.18	0.9495	11.981*	.638**
8. I could teach a robot something if I would try hard enough.	4.08	1.0875	9.951*	.550**
9. I could easily learn how to use a robot.	4.31	0.9675	11.436*	.653**
10. I could teach a robot to complete easy tasks.	4.36	0.898	10.401*	.610**
11. If I would use a robot, I would always know how and why it behaves like it does.	3.84	1.074	10.049*	.595**
12. I could do easy adjustments on a robot by myself.	3.53	1.061	12.44*	.653**
13. I could use a robot in daily life.	4.44	0.9395	10.776*	.631**
14. I would feel comfortable while interacting with the robot.	4.24	0.888	9.973*	.576**
15. If a robot is doing something wrong, I could find a way to change its behavior.	4.085	1.071	11.131*	.594**
16. Robots are easy to control.	3.54	1.107	9.842*	.596**
17. I could deploy a robot in a specific way to save time.	4.045	1.0065	12.867*	.689**
18. I am very confident in my abilities to control a robot.	3.77	0.9685	14.663*	.727**

^*^p < 0.05, ^**^p < 0.01

### Principal Component Analysis (PCA)

The Bartlett Sphericity Test and Sample Suitability Test (KMO) were carried out to assess the suitability of the data for component analysis (see Table 3). Bartlett's Sphericity Test reached statistical significance (*χ*^2^ = 2189.567, *df* = 153, *p* < 0.001), and KMO value was 0.906, indicating the data were suitable for factor analysis. Further, parallel analysis suggested extracting 2 factors as optimal (Hayton et al., 2004). Additionally, factor extraction was performed using the principal component method with a fixed number of factors at 2. Criteria for item exclusion included: (1) commonality below 0.3 (Ul Hadia et al., 2016), (2) factor loadings less than 0.4 (Costello & Osborne, 2005), (3) identical coefficients with loads equal to or greater than 0.4 for the various main components (Schönrock-Adema et al., 2009), and (4) factors with fewer than three items remaining after exclusion (Costello & Osborne, 2005). In the first principal component analysis (PCA), items Q3, Q11, Q12, Q13, and Q18 were excluded. The second PCA on the remaining thirteen items revealed a robust two-dimensional structure.

**Table 3:** Results of principal component analysis

	Factor1	Factor2	commonality
1. I could set up a robot according to my wishes and my environment. 我可以根据自己的意愿和环境设置机器人。	0.788		0.648
2. I could get a robot to perform a specific task. 我可以让机器人执行特定的任务。	0.784		0.637
4. I think I could adjust a robot the way that it could help me in my daily life. 我认为我可以调适机器人从而让它在日常生活中帮助我。	0.573		0.451
8. I could teach a robot something if I would try hard enough. 如果我足够努力，我可以教机器人一些东西。	0.643		0.430
10. I could teach a robot to complete easy tasks. 我可以教机器人完成容易的任务。	0.620		0.461
15. If a robot is doing something wrong, I could find a way to change its behavior. 如果机器人做错了什么，我可以想办法改变它的行为。	0.562		0.385
17. I could deploy a robot in a specific way to save time. 我可以用特定的方式配置机器人，以节省时间。	0.666		0.533
5. It is easy to use a robot. 机器人很容易使用。		0.756	0.592
6. If I should solve a problem with the assistance of a robot, I could do that. 如果我可以有机器人的协助，我就能解决问题。		0.623	0.413
7. To achieve a specific goal with the assistance of a robot will not be a problem for me. 在机器人的协助下实现特定目标对我来说不成问题。		0.663	0.515
9. I could easily learn how to use a robot. 我可以轻松学会如何使用机器人。		0.688	0.537
14. I would feel comfortable while interacting with the robot. 在与机器人交互时，我会感觉很舒适。		0.571	0.397
16. Robots are easy to control. 机器人很容易控制。		0.677	0.497
Eigenvalue	5.156	1.340	
Variance contribution rate (%)	26.283	23.684	
Cumulative variance contribution rate (%)	26.283	49.967	

### Confirmatory Factor Analysis (CFA)

**Fig. 2:**
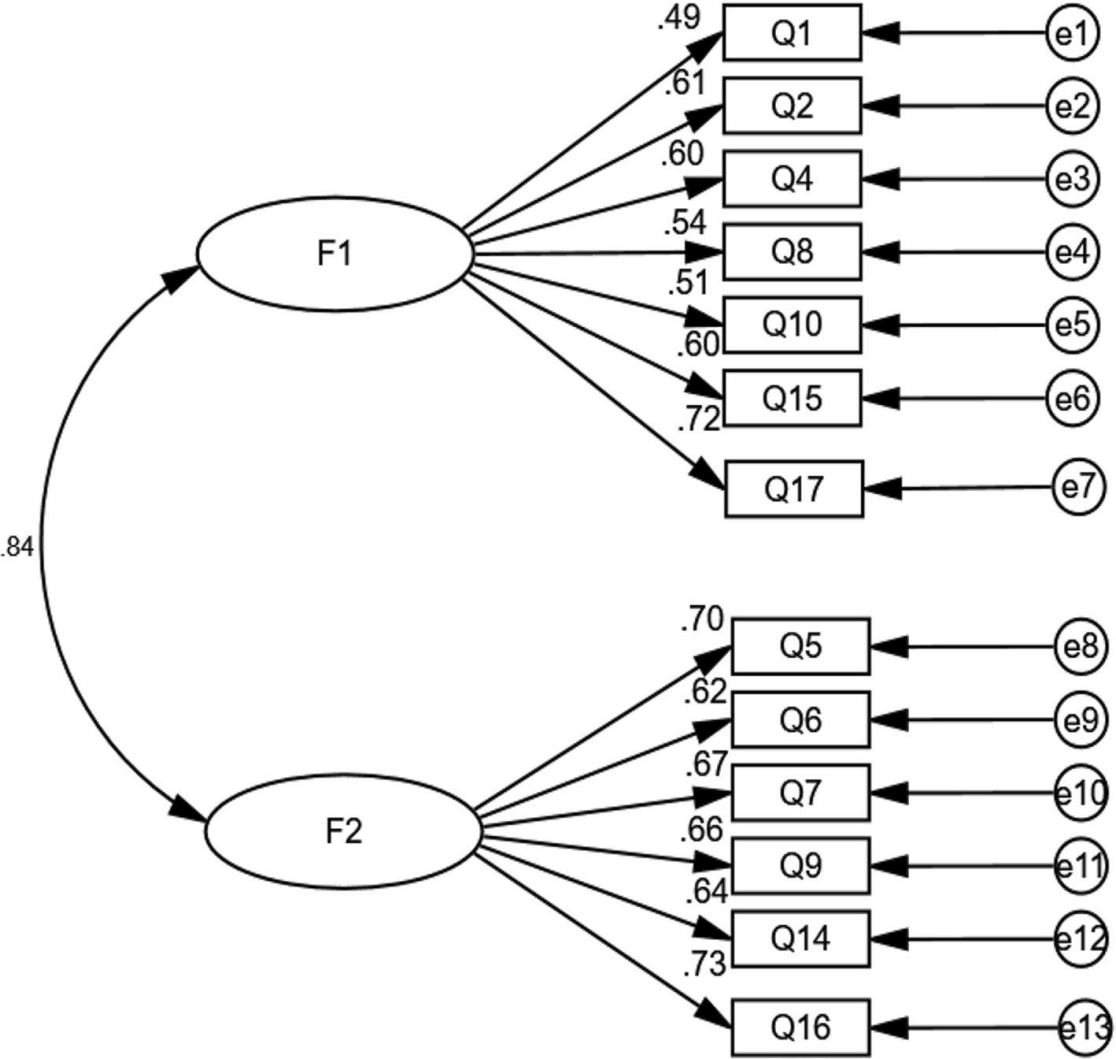
Results of CFA

To verify the validity of the Chinese SE-HRI scale, that is, to check alignment between the conceptual and the actual models, as well as the item-factor relationships, confirmatory factor analysis (CFA) was performed (see Fig. 2). The results showed that *χ*^2^/*df* = 4.289, GFI = 0.919, CFI = 0.901, and RMSEA = 0.081, indicating that the Chinese SE-HRI scale demonstrated robust construct validity (Hu & Bentler, 1999; Marsh & Hocevar, 1985).

### Criterion-related validity

Correlation analysis was employed to establish the criterion-related validity of the Chinese SE-HRI scale. It was found that both General Self-Efficacy and Willingness to Accept the Use of artificial intelligence (AI) were positively correlated with self-efficacy in HRI (*p*s < 0.01), while Negative Attitudes toward Robots showed an inverse correlation (*p*s < 0.01), proving excellent criterion-related validity of the Chinese SE-HRI scale (see Table 4).

**Table 4:** Results of criterion related validity

Variables	M	SD	1	2	3	4	5
1. Chinese SE-HRI	4.48	0.695					
2. F1	4.57	0.692	.901**				
3. F2	4.38	0.823	.931**	.682**			
4. General Self-Efficacy Scale	2.83	0.605	.629**	.569**	.583**		
5. Willingness to Accept the Use of AI Devices Scale	4.14	0.514	.582**	.512**	.552**	.387**	
6. Negative Attitudes toward Robots Scale	2.57	0.744	-.565**	-.481**	-.549**	-.417**	-.400**

^**^p < .01

### Reliability analysis

The internal consistency reliability and odd–even split-half reliability of the scale were analyzed (see Table 5). The results indicated that the Chinese SE-HRI scale demonstrates robust reliability (Tavakol & Dennick, 2011).

**Table 5:** Reliability indicators of the SE-HRI scale

Reliability indicators	F1	F2	Total scale
Internal consistency reliability (α)	0.781	0.829	0.876
Odd–even split-half reliability	0.786	0.854	0.863

## Discussion

This study intends to validate the SE-HRI as a measure of human–robot interaction within the context of Chinese culture. Through PCA, CFA, criterion-related validity analysis, and reliability analysis, the scale has been demonstrated to be an effective and reliable tool for assessing human–robot interaction in Chinese adults.

The results of PCA, CFA, criterion-related validity analysis, and reliability analysis indicated that the scale achieved good reliability and validity, effectively measuring the concept of self-efficacy in human–robot interaction. It may be attributed to the standardized translation process used in the study (Guo et al., 2020), enhancing participants’ accurate understanding of the item content. However, it is noteworthy that the English version of the scale is unidimensional, whereas the Chinese version in this study is two-dimensional. One possible explanation for this discrepancy is the procedure used during the survey administration. When we asked participants to fill out the questionnaires, they were first shown a picture of the robot and then asked to imagine interacting with it. Such an operation would make their image of the robot more concrete, their understanding of self-efficacy more precise, and their perception and dimensional distinction of the robot more detailed, resulting in a two-dimensional structure. This discrepancy could be linked to variations in individuals' perceptions of robotics (Lim et al., 2021). When participants are less familiar with robotics, they tend to have a generalized understanding, leading to a unidimensional perception. As the growing development of China's robotics industry progresses, Chinese adults are becoming increasingly familiar with robots, allowing for a more nuanced perception and differentiation of dimensions.

In addition, there was a significant positive correlation between the general self-efficacy scale, the Willingness to Accept the Use of AI Devices and the Chinese SE-HRI. Conversely, the Chinese SE-HRI was significantly negatively correlated with the scores of the negative attitudes toward robots scale. These findings indicate the robust criterion-related validity of the Chinese SE-HRI scale, aligning well with self-efficacy and willingness to accept the use of AI devices in general (Latikka et al., 2019). In other words, higher levels of self-efficacy correspond to an elevated SE-HRI and a greater inclination to accept and use robots. This inclination may stem from the belief that individuals with high self-efficacy will perform well and adapt effectively when interacting with robots, fostering a willingness to use them (Pasparakis et al., 2023). Furthermore, individuals with high self-efficacy in HRI tend to exhibit positive attitudes toward robots, while those with low self-efficacy in HRI demonstrate negative attitudes. One plausible explanation is that negative attitudes diminish an individual's self-efficacy, and concurrently, low self-efficacy further reinforces negative attitudes, that is, negative attitudes and low self-efficacy tend to interact in a vicious cycle (Hampel et al., 2023). In summary, the Chinese SE-HRI scale proves to be a reliable and effective tool for evaluating self-efficacy in human–robot interaction, providing a solid foundation for research in this filed within the Chinese context.

There are two primary contributions in this research. Firstly, the Chinese SE-HRI scale emerges as a reliable and valid assessment tool for the field of Human–Robot Interaction (HRI) in China. The establishment of the Chinese SE-HRI scale contributes to the burgeoning artificial intelligence and technology industry in China, ensuring that subsequent advancements in robot technology align more closely with the needs and preferences of the Chinese population. Moreover, it plays a crucial role in enhancing the design and performance of human–robot interaction systems. Essentially, this study holds great importance to the understanding of self-efficacy in human–robot interaction and can facilitate cross-cultural comparative studies. Secondly, this study highlights that the General Self-Efficacy, the Willingness to Accept the Use of AI Devices, and the Negative Attitudes towards Robots are effective criteria for SE-HRI, demonstrating the criterion validity of SE-HRI and supporting the empirical correlation of these variables.

When interpreting the findings of existing studies, it is crucial to acknowledge certain limitations. Firstly, the Cronbach's alpha of the Willingness to Accept the Use of AI Devices Scale is 0.629, which falls short of the standard typically used to indicate good internal consistency. Future studies could improve the better assessment of the reliability of measurement tools’ reliability and avoid the excessive influence of single indicators on research findings, thus improving the validity and reliability of the findings. Moreover, the predominant age range of participants falls between 19 and 25 years old, highlighting the necessity for broader age representation for a more comprehensive understanding in future studies. Secondly, the prevalence of robots varies across different regions in China (Wang et al., 2018). Future research could address this by conducting region-specific investigations, accounting for the diverse rates of robot integration in different regions. In our study, we were unable to systematically assess measurement invariance between the original and translated versions due to resource constraints and the unavailability of English language samples. Assessing measurement invariance is critical to ensuring the validity of the translated version of the scale, and future research could systematically assess measurement invariance between the original and translated versions by recruiting a sample of native English speakers. This would provide a more comprehensive understanding of the applicability and reliability of the translated versions of the scales in different cultural and linguistic contexts..Finally, the data provided in this study are all self-reported, subject to the influence of social expectations and personal subjective biases (Anvari et al., 2023). Further research could incorporate behavioral observations to obtain a more nuanced and objective perspective.

## Conclusion

This study aims to translate the SE-HRI and investigate its reliability and validity through a large sample of Chinese adults. The findings revealed that the Chinese SE-HRI scale contains 2 dimensions and 13 items, meeting the measurement criteria with good reliability and validity. This is the first application of the self-efficacy in HRI with Chinese adults, thus expanding the utilization of the SE-HRI scale in Chinese culture.

## Data Availability

The data that support the findings of this study are available on request from the corresponding author. The data are publicly available. We have uploaded the data to https://osf.io/wvdmf/. The DOI number for the data is 10.17605/OSF.IO/WVDMF.

## Abbreviations

HRI: Human–robot interaction
SE-HRI: Self-Efficacy in Human–Robot Interaction
AI: Artificial intelligence
GSES: General Self-Efficacy Scale
NARS: Negative Attitudes toward Robots Scale
EFA: Exploratory factor analysis
CFA: Confirmatory Factor Analysis
